# RETRACTION: Loss‐of‐Function CARS1 Variants in a Patient With Microcephaly, Developmental Delay, and a Brittle Hair Phenotype

**DOI:** 10.1002/mgg3.70301

**Published:** 2026-09-11

## Abstract

**RETRACTION**: C. Del Greco, M. E. Kuo, D. E. C. Smith, M. I. Mendes, G. S. Salamons, M. Nemcovic, R. Kodrikova, S. Sestak, M. Stancheva, and A. Antonellis, “Loss‐of‐Function CARS1 Variants in a Patient With Microcephaly, Developmental Delay, and a Brittle Hair Phenotype,” Molecular Genetics & Genomic Medicine 13, no. 2 (2025): e70078, https://doi.org/10.1002/mgg3.70078.

The above article, published online on 18 February 2025 in Wiley Online Library (https://onlinelibrary.wiley.com/), has been retracted by agreement between the authors; the journal Editor‐in‐Chief, Paraminder Dhillon; and Wiley Periodicals, LLC. The retraction has been agreed upon due to the lack of appropriate authorization for the publication of the CARS1 variants related to the specific patient described in this clinical report. In addition, written consent for publication was not obtained from the child's legal guardian.

**RETRACTION**: C. Del Greco, M. E. Kuo, D. E. C. Smith, M. I. Mendes, G. S. Salamons, M. Nemcovic, R. Kodrikova, S. Sestak, M. Stancheva, and A. Antonellis, “Loss‐of‐Function CARS1 Variants in a Patient With Microcephaly, Developmental Delay, and a Brittle Hair Phenotype,” Molecular Genetics & Genomic Medicine 13, no. 2 (2025): e70078, https://doi.org/10.1002/mgg3.70078.

The above article, published online on 18 February 2025 in Wiley Online Library (https://onlinelibrary.wiley.com/), has been retracted by agreement between the authors; the journal Editor‐in‐Chief, Paraminder Dhillon; and Wiley Periodicals, LLC. The retraction has been agreed upon due to the lack of appropriate authorization for the publication of the CARS1 variants related to the specific patient described in this clinical report. In addition, written consent for publication was not obtained from the child's legal guardian.

